# Perceived barriers and opportunities of providing quality family planning services among Palestinian midwives, physicians and nurses in the West Bank: a qualitative study

**DOI:** 10.1186/s12913-024-11216-4

**Published:** 2024-07-09

**Authors:** Sahar Hassan, Hadeel Masri, Isra’ Sawalha, Berit Mortensen

**Affiliations:** 1 Department of Nursing and Master program of Women’s Health, Faculty of Pharmacy, Nursing and Health Professions, Birzeit University, Birzeit, Palestine; 2 Women’s Health and Development Unit, Ministry of Health, Ramallah, Palestine; 3 Master program of Women’s Health, Faculty of Pharmacy, Nursing and Health Professions, Birzeit University, Birzeit, Palestine; 4 Faculty of Health Sciences, Oslo Metropolitan University, Oslo, Norway

**Keywords:** Family planning, Barriers, Midwives, Palestine

## Abstract

**Background:**

Despite advancements in family planning (FP) services, several barriers persist in the Occupied Palestinian territory (oPt), blocking women’s access to suitable, high-quality and equitable FP services. The aim of this study was to understand how healthcare providers perceive their abilities, barriers and opportunities in providing good quality FP services. Furthermore, it seeks to explore knowledge and training regarding FP among healthcare providers engaged in providing FP services.

**Methods:**

A qualitative study was undertaken from August to September 2022 in seven Primary Health Care (PHC) clinics distributed in three governorates and operating under the Palestinian Ministry of Health (MoH) in the West Bank. Semi-structured, in-depth face-to-face interviews were conducted with 13 health providers (Physicians, midwives and nurses), using an interview guide in Arabic language. Transcripts were subsequently analyzed using the six phases of reflexive thematic analysis.

**Results:**

FP services face various challenges, including shortages in resources such as staff, supplies, infrastructures and FP methods. Midwives possess significant potentials to offer accessible, high-quality, efficient and equitable FP services, yet, their capacities remain underutilized, representing a missed opportunity for a country like Palestine. The study provided a current overview of FP services while illustrating the need for quality FP services and the need for an updated continuous education and training, updated standardized guidelines and protocols and supportive supervision are needed across all levels of healthcare providers. Finally, providers reported a wide range of structural barriers to FP services.

**Conclusions:**

It is crucial to meticulously address both community-related and health system factors to enhance the fulfillment of FP needs and reduce unintended and closely spaced pregnancies. Policymakers should invest in the development of laws and regulations regarding FP services, promoting a comprehensive and holistic approach to FP services. This includes formulating supportive policies, capacity building of human resources and maintaining security of FP commodities.

**Supplementary Information:**

The online version contains supplementary material available at 10.1186/s12913-024-11216-4.

## Introduction

Sexual and Reproductive Health and Rights (SRHR) constitute an integral component of the Sustainable Development Goals (SDGs), specifically goals three, four, and five. These goals were declared to contribute to the sustainable advancement of nations [1, 2]. However, significant disparities in SRHR indicators were noted between low income and high-income countries. For example, variations in unintended pregnancies rates indicate gaps in access to family planning (FP) services [2]. This necessitates the need for more investment to ensure equitable access to high quality sexual and reproductive health care (SRH) services [2, 3]. FP is a well-documented cost-effective intervention that can prevent not only unintended pregnancies, but also can preserve women’s lives. There is clear evidence of the positive effects of FP on promoting women’s health by reducing maternal and infant mortality and morbidity [4]. Moreover, the use of FP is associated with reduced poverty rates and better economic conditions for families. It also leads to fewer unsafe abortions, better educational opportunities, better quality of life and empowerment for women [2, 5, 6].

Despite the decline in total fertility rate in the Occupied Palestinian territory (oPt) during the past decade, it is still high (3.8 per woman) [7] compared to other Arab countries; Jordan 2.7, Lebanon 2.1, Egypt 3.3, Yemen 3.7 [8]. The contraceptive prevalence rate is 57.3% among currently married Palestinian women age 15–49 who are using any contraceptive method [7]. This is a little higher than the contraceptive prevalence in the Eastern Mediterranean Region (EMR); 48% [9] and lower than Jordan (61%), Morocco (67.4%) and Tunisia (64.4%) [10]. In Palestine, the highest reported usage was IUD 26.1% followed by pills 6.9% among the modern contraceptive methods [7]. The unmet need for FP for married women aged 15–49 is 12.9% (13.6% in the WB and 11.9% in Gaza) with the highest rates for women in rural areas (14.8%) [7] and could be high among poorest population [10].

In the oPt, FP services are delivered by various health providers including; the Ministry of Health (MoH) as the primary healthcare service provider, United Nations Relief and Works Agency (UNRWA) which addresses the needs of Palestinian refugees affected by the 1948 and 1967 wars, non-governmental organizations and the private sector. According to the MoH annual report 2019, FP services were provided in 281 governmental primary healthcare centers (PHC) in the West Bank. The highest reported FP methods are pills 67.1%, IUDs 17.4% and condoms 12.2% [11]. Women are required to pay fees to access FP services at the MoH clinics, whether they are insured or not. These fees include charges for registration and file opening, laboratory tests, physician visit, medication to treat any condition, and the FP method itself. While individually these fees may seem small amount, collectively they amount to cost that constitutes a burden for many women.

Very few studies have been conducted during the past decade on FP in Palestine. A recent cross-sectional survey among refugee women attending UNRWA clinics found that refugee women rely on UNRWA clinics to receive FP methods and a higher use of modern methods among women aged 35 and above [12]. Few studies from Gaza highlighted the issue of unintended pregnancies [13] and barriers to FP methods use among women [14]. Misconceptions, fears, supply shortages were barriers to FP use reported by women and their healthcare providers from Gaza [14]. A randomized control study (2019) concluded that using mobile phone text messages may improve attitudes knowledge-perceived norms and intention to use of FP methods among young women in Palestine [15]. A recent qualitative study conducted in both West Bank and Gaza regions of Palestine explored obstacles and facilitators encountered by healthcare providers while providing long-lasting reversible contraceptive methods (LARCMs) [16]. The study revealed numerous challenges facing the delivery of LARCMs including insufficient training and experience among healthcare providers, lack of knowledge about contraception among women, as well as shortages and high cost of FP methods.

In the oPt, FP methods are provided free of charge for refugees and at reasonable cost for those covered by government insurance. In a 2019 study, inadequate evidence-based FP training guidelines and material, poor FP counseling provided during preconception care, and lack of regular monitoring and reporting of evidenced-based indicators were reported from countries in the Eastern Mediterranean Region (EMR) [9]. Additionally, current data on access and sustainability of FP services is not available and data on quality, practices of FP services and competencies of health care providers have never been studied. The aim of this study is to understand how healthcare providers (midwives, physicians and nurses) perceive their abilities, barriers and opportunities in providing good quality FP services. Furthermore, it seeks to explore knowledge and training regarding FP among healthcare providers engaged in providing FP services.

## Methods

### Study design

A qualitative study design, using individual in-depth interviews was utilized to gain deeper understanding and insight into healthcare provider’s perspectives on FP services within the Palestinian context.

### Study setting

The study was carried out in various governorates of the West Bank, oPt. Health care providers from PHC clinics operating under the Palestinian MoH in the north, middle and south governorates in the West Bank and representing urban, rural and camp contexts, were invited to participate.

### Recruitment and data collection

A purposive sampling approach was used to select heterogeneous sample of clinics and participants, to ensure maximum variation among sites and healthcare providers including; midwives, nurses and physicians who are actively involved in FP services [17]. Face-to-face semi-structured in-depth interviews, was conducted between August and September 2022, by a research assistant (RA) a midwife and master student (IS), who had been trained in qualitative interview technique. A total of seven clinics were targeted for data collection because they provide FP services, (four in north, two in middle, and one in the south). (Table 1) The selection of the seven clinics depended on their location in various Palestinian contexts, within different governorates of the West Bank and the type of healthcare providers in the clinic; clinics that were staffed with at least one midwife were selected. After obtaining formal permission from the MoH, contacts were established with potential participants who were eligible about the aims of the study and to ensure they were available in the clinic on the scheduled day for the interview. We considered all healthcare providers who participate in providing FP services in the selected clinics as eligible including midwives, physicians and nurses.

To ensure relevance, completeness, quality and content, the first two interviews were transcribed, carefully read and discussed among an experienced researcher (SH) and RA (IS). Gaps, clarity of responses and the need for more information on certain responses were identified and discussed among researchers to be focused on during the next interviews. SH and IS discussed the context of interviews, main points and notes on the site throughout the process of data collection for each two to three interviews to ensure rich data. Thirteen individual interviews were conducted with care providers comprising of midwives, nurses and physicians, at time convenient to the participant.

A topic guide for the interviews was prepared, discussed and revised among all authors (see Additional file 1). It was built based on the researchers’ knowledge and expertise of FP services in Palestine and the context of Palestinian midwives. The following topics were covered: challenges and facilitators related to education, training and practice faced by healthcare providers while providing FP services and suggested solutions and strategies from the participant’s perspectives. The interviews started with questions related to participant’s background, education and work experience. Two authors are clinicians (SH and HA) with extensive experience in the context of women’s health services in Palestine. SH is an experienced nurse-midwife leader and an academic, familiar with context of Palestinian midwifery education and scope of practice. HA is an Obstetrician and Gynecologist and head of the Women’s Health Unit at the MoH and familiar with context of FP services in Palestine. The interviews were performed using open-ended questions to allow participant’s’ maximum elaboration. Follow up and probing questions were also used to elicit clear details and understanding, such as “can you tell me more…” “can you give me an example.”.

The length of the interviews varied between 20 and 57 min (Average 38 min). All interviews were conducted in Arabic language, voice recorded except one refused to be recorded; hand-written notes were done during the interviews. No one refused to participate or withdrew before finishing the interview.

**Table 1:** The selected governorates and types of clinics

Governorates	Number and type of clinics	Number of participants
North	4 (one urban, one rural, 2 camp)	7
Middle	2 (one urban, one rural)	3
South	1 (rural)	3

### Data analysis

All interviews were transcribed into Arabic verbatim and were anonymized by giving each interview a serial number from 1 to 13. The data were analyzed inductively using reflexive thematic analysis applying the six phases recommended by Braun and Clarke [18] including: (1) familiarization with data during and after each interview and transcription, (2) systematic coding, (3) developing initial themes from collated data, (4) reviewing themes, (5) defining and naming themes, and 6.writing a report [18]. The first and third authors became familiar with the data by listening to interviews immediately after conducting 1–2 interviews, reading transcripts, discussing content, and developing initial codes. After finishing all interviews, a plan of analysis was shared and discussed among the three Arabic speaking authors (SH, HM, IS), who carefully read the transcripts several times individually and making notes on first impressions as a first step, then independently developed codes from the content of transcripts by careful reading and coding line-by-line. After that, the three authors (SH, HA, IS) reviewed and discussed initial codes, collated data across the various interviews to prepare development of initial sub-themes. Table 2 illustrates examples of data extraction from original transcripts containing units of data and line-by-line coding. Codes and collated data were extensively reviewed several times for patterns and similarities across interviews, developed into initial sub-themes and translated to English (SH, HA, IS) [19] as illustrated in Appendix 1. This approach was applied by each researcher, in each separate interview then similar sub-themes from each interview were aligned for discussions to define and name main themes. We used an inductive approach to open for important information beyond our research question. The translated collided data and sub-themes were shared with the English-speaking authors (BM), who is familiar with the Palestinian context from working with maternal health projects in the oPt for many decades. The translated collated data and sub-themes were extensively discussed, revised, and modified among the three researchers, checked back with original data and against each other and named [19]. (Appendix 2) Codes, sub-themes and initial themes were discussed and revised multiple times among researchers (SH, HA, IS & BM) until a consensus that reflected and was supported by the original data. The analysis was done manually using hard and soft copies of the transcripts.

The reflexive analysis in developing codes, sub-themes and themes included fruitful discussions among the research team, involving various experience and understanding of how we could develop themes from the data as guidance to improved contraceptive care. Throughout extensive review of transcripts, suitable quotations were selected and translated into English.

### Ethical considerations

Ethical approval for the study was obtained from the Ethical Research Committee at the Faculty of Pharmacy, Nursing and Health Professions, Birzeit University, West Bank (BZUPNH2134). All necessary administrative permissions were obtained from stakeholders at the MoH. We adhered to the ethical standards in carrying research on humans. Informed consent was obtained. Confidentiality of participants was ensured as no names and identifiable data as age, employee number, IDs or phone numbers were mentioned in voice records or transcripts. Transcripts were given serial numbers.

## Results

### Characteristics of the study participants

Thirteen participants, aged between 35 and 58, participated in the study. Among these participants, eight were midwives, two were nurses, and three were physicians, with years of experience ranging from 12 to 36 years. Table 2 summarizes the participants characteristics.

**Table 2:** Characteristics of health providers

Variable	Number
Age	
(Average, range) years	(43, 35–58)
Specialty	
Physician	3
Midwife	8
Nurse	2
Location of clinic	
North	4
Middle	2
South	1
Years of experience (Average, range) years	(19.5, 12–36)
FP education and training	
Yes	6
Yes, partial*	5
No	2

*Partial: Few lectures on Family Planning (FP) were integrated into other courses, without or inadequate clinical training, as noted by Physicians and midwives

The main themes and sub-themes are presented in Table 3. Our thematic analysis revealed three themes: midwives in the context of FP, landscape of FP services, and perceived barriers to FP services. Each theme included subthemes. Eleven subthemes were identified.

**Table 3:** Themes and subthemes

Themes	Subthemes
Midwives in the context of FP	1) Midwives’ potentials not optimized Education, training and Knowledge Midwives’ scope and role in FP services. 2) Midwives’ perceptions of contraceptive methods and rights
Landscape guide for quality FP services	1) FP service provided by incompetent staff Nurses’ education and role Updated continuous education and training needed at all levels 2) Routines and protocols 3) Huge demand for structured supervision, education and updates 4) Good quality is a lucky draw Availability of contraceptive methods 5) Suggestions for improvements
Structural barriers to FP services	1) COVID negative effect on FP services 2) Providing FP services with empty hands Accessibility to FP methods Structural and disposals deficiencies 3) Context and cultural barriers

### Theme 1: midwives in the context of FP

Midwives are well-prepared to actively engage in the delivery of FP services. However, their scope of practice is restricted. Two sub-themes emerged from the midwives in the context of FP.

### Subtheme 1: midwives’ potentials not optimized

This sub-theme presents findings related to midwives’ education, training, knowledge, and scope of practice. Nearly all midwives received basic education as a FP course as part of their basic midwifery education (Table 1). Some midwives reported that their clinical training during the FP course was inadequate. Very few reported that they received additional training during work, but this training did not authorize them to provide FP services.

*“Of course, there is one complete course in the midwifery college about family planning.”* (P7, midwife).

Despite their educational preparation, midwives reported that their role in FP services is limited. Their roles include receiving women, measuring vital signs, starting a medical record, and counseling women about desired and available FP methods.

*“The woman comes to us, we measure her vital signs, we ask her, and we explain to her about the contraceptive methods we have. After she chose a method, she sees the physician, and we explain to the physician what the woman chose.”* (P7, midwife).

FP services, including IUD insertion, are part of the scope of the midwives work in the United Nations Relief and Works Agency (UNRWA) primary clinics. In contrast, midwives who work in the governmental sector are not authorized to provide FP services. Some midwives expressed disappointment due to limitations in their scope of practice, although they are the only trained healthcare providers who exist in the clinic on a daily basis. Physicians also reported that it is not allowed that a midwife insert an IUD.

*“We, as midwives, this is our job. Why don’t we receive training to insert the IUD like midwives at the UNRWA? We were trained that inserting an IUD is part of the midwife’s role, for instance, here at the PHC, it is not allowed.”* (P2, midwife).

*“What does that mean? They didn’t insert an IUD, nor did they give anything. It is prohibited.”* (P10, physician).

*“I don’t understand, they trained me to insert an IUD, and you didn’t allow me to do it. Why did you train me then?”* (P9, midwife).

### Subtheme 2: midwives’ perceptions of contraceptive methods and rights

Some midwives lacked updated training and revealed certain perceptions or misconception and may lack proper knowledge regarding FP services and methods, which can potentially hinder women’s access to effective contraception. For instance, some midwives may refrain from providing counseling on contraceptive methods if a woman’s husband is not present, or may exclusively promote natural methods that have a higher risk of user error. These practices can result in women missing out on the opportunity to access reliable FP methods or continuing to rely on unreliable methods, which may lead to unintended pregnancy.

*“When the husband is with his wife, you can talk to him. Sometimes you cannot advise her to use family planning methods without her husband’s knowledge”.* (P3, midwife)

*“I’m convinced that the natural is better; for some women, the IUD is better. IUDs are better, but if the woman uses a natural methos like breastfeeding, of course, I will definitely support her. I will definitely tell her that breastfeeding positively affects her and the child’s health. If she comes to tell me that her husband ejaculates outside the vagina, and both are 100% okay and comfortable for 5 years, why to tell her to use other methods?” (*P4, midwife).

### Theme 2: Landscape guide for quality FP services

This theme introduced us to deep and comprehensive insight into the current landscape of FP services. Within this overarching theme, five distinct sub-themes emerged (Table 5). The MoH’s FP services are primarily staffed by nurses, midwives, and physicians. Among these, physicians are the primary providers of contraceptive methods, while nurses and midwives play a supporting role in delivering this essential service. Participants have provided detailed accounts of how FP services are executed in various clinics, including aspects such as daily routines, service quality, the presence of supervision and technical guidance, the range of available contraceptive methods, and, lastly, their perspectives on potential improvements of these services.

#### Subtheme 1: FP service provided by undertrained staff

##### Nurses’ education and role

Many primary healthcare clinics that offer FP services are staffed by nurses. Nurses reported that they had little or no basic education in FP and they had not undergone training in FP. However, sometimes they received some general on-the-job training on FP upon starting their work in the FP services.

*“We learned about the side effects of family planning methods and their effectiveness. As nurses, we do not delve into this subject as much as midwives.”* (P12, nurse).

*“I did not take too many of these courses because I am not a midwife. I am a nurse. But we received training on pregnancy and family planning, how to insert an IUD, and the available family planning methods in the Ministry of Health.”* (P5, nurse).

Nurses have indicated that they have cultivated expertise through their work experience and exposure to various FP cases. Over time, their involvement in this field has deepened their comprehension of different methods, their mechanisms, effectiveness, and potential complications. Nevertheless, it is evident that they have gaps in their knowledge when they discussed specific FP methods. Their responsibilities encompass welcoming the woman, initiating her medical record, and recording vital signs such as blood pressure and weight.

*“We, nurses, open the file for the woman; take personal information, and measure weight, height, blood pressure.”* (P12, nurse).

*“When I started working, I was exposed to women who came for this service and learned on my own and by experience. For example, the IUD is suitable for some women, while pills are suitable for others.”* (P5, nurse).

##### Updated continuous education and training needed at all levels

Physicians reported that they were introduced to FP knowledge during their medical education, but formal training in this area was absent from both their basic medical education and on-the-job training. Physicians are the primary providers responsible for offering all available FP methods. Some physicians mentioned that they made efforts to self-educate by shadowing colleagues when possible. Conversely, others expressed challenges in gaining expertise in areas such as birth control pill usage and associated complications or in the insertion of intrauterine devices (IUDs), where they preferred having ultrasound equipment available in the clinic to ensure accurate IUD insertion. Midwives reported that a group of highly skilled female physicians had retired without passing on their knowledge and skills.

*“She is a senior physician, and she trained me, and by virtue of experience, I refer to her as we do not have a reference.”* (P6, physician).

*“The biggest challenge for me is oral contraceptives, and so far, because it is a wide topic and you always need a guide. Sometimes I need a reference.”* (P6, physician).

*“I prefer the family planning room has an ultrasound, so that the doctor can install the IUD under ultrasound guidance with more control, see how the IUD is being inserted, and the success rate will be higher.”* (P10, physician).

*“Before 2002, there was a retirement for trained female doctors. They did not train new female doctors who would replace those retired female doctors.”* (P8, midwife).

### Subtheme 2: routines and protocols

The majority of healthcare providers reported either the absence of established work protocols or having protocols in the form of announcements or information disseminated during monthly administrative meetings.

Contraceptive methods are administered by physicians based on factors such as their availability, the woman’s preferences, and suitability for her specific medical condition. Providers mentioned adhering to “known” protocols and practices related to contraceptive methods, which are based on their foundational education. For example, they adhere to aseptic techniques when inserting intrauterine devices (IUDs) and refrain from inserting an IUD for a woman with anemia or prescribing combination oral contraceptive pills for recently delivered women who are breastfeeding or have coagulation issues, or prescribing mini-pills for non-breastfeeding women. Midwives and nurses reported that they counsel women on the various contraceptive options available and help them make informed decisions based on their preferences.

*“For pills, we tell the woman if she has a problem with pressure, coagulation problems, or has had a previous pulmonary embolism, she is not allowed to use pills. She may use an IUD or condoms”.* (P11, midwife)

*“We give progesterone only pills, after the woman gives birth. Then, by 7 to 8 months, when she starts feeding the baby solid food, we convert her to compound pills, as progesterone-only pills will not be effective. If she wants an IUD, we install it on the 35th day or on the fifth or sixth day of her period. If her cycle is short and light, she can come any day to have an IUD inserted”.* (P9, midwife)

### Subtheme 3: huge demand for structured supervision, education and updates

Providers reported that supervision is generally minimal and restricted in the form of administrative supervision rather than technical guidance. Some providers participate in routine monthly administrative meetings where they address administrative matters and supervisors convey any new updates or instructions related to their practice. On the other hand, providers working in clinics situated in remote areas expressed dissatisfaction with the infrequent and inadequate supervision they receive. In such locations, supervision reported to be scarce, with periods of absence lasting for several months or even more than a year.

*“Let me tell you, here in the south area, we are neglected by the ministry. Not many officials come to follow up with us, visit the site, or anything like that. Maybe it’s been a year or two since someone from the ministry came to check on us. We submit monthly reports to the ministry”.* (P12, nurse)

*“This monthly meeting is held every month or every 2–3 months according to the need nurses where they receive updates, i.e., a lecture on quality. Now we have a continuing education section, where we assess the needs and priorities and request them, i.e., infection control, on the 14th of each month”. (P8, midwife)*

### Subtheme 4: good quality is a lucky draw

Providers’ opinions varied regarding the quality of the FP services they provide. Some providers noted that the quality was poor, attributing it to frequent and prolonged interruptions in the availability of contraceptive methods at the clinic, as well as the inconsistent presence of physicians on a daily or regular basis. These interruptions have had negatively impacted the reputation of FP services within the local communities, eroding the trust that women have in the clinic and causing them to discontinue seeking services there. Conversely, other providers regarded the quality of FP services as adequate or satisfactory.

*“When the doctor comes to me once a month and I want to insert an IUD, you know the fifth day of the start of menstruation is optimal. But if the woman’s period arrives on the 5th and the doctor visits on the 16th, 17th, or 19th, as you can see, how can I effectively provide IUD insertion within the Ministry of Health? In this case, people go outside to have the IUD inserted.”* (P4, midwife).

Providers emphasized that there has been a significant change from the past routines of FP services. Previously, these services were available on a daily basis, offering all methods provided by the United Nation Population Fund (UNFPA), and they were entirely free of charge for everyone. Now, the MoH purchases FP methods to supply clinics for contraception use. Many providers have reported a substantial decline in the number of beneficiaries in recent years.

*“The doctor who used to work with me used to see 40 women for IUD service each day. Now, I don’t know how many women they see. But the numbers are very few.”* (P8, midwife).

#### Availability of contraceptive methods

Healthcare providers reported that contraceptive methods are not consistently available in clinics. Availability varies from one clinic to another, with certain methods being present in some clinics but absent in others.

*“There’s nothing here, I mean, there’s barely anything available besides pills. Let’s say condoms are available occasionally (sarcastically gestures with her hand on her face). IUDs aren’t even provided here. The IUD service isn’t offered because the village is very small”.* (P4, midwife)

Certain methods may remain unavailable for several months or even years. Providers explained how this interrupted availability of contraceptive methods negatively affects women’s access to services. They noted that when women repeatedly visit the clinic without being able to obtain their desired contraceptive method, they eventually stop seeking services at the clinic. Some women may opt to obtain contraceptive methods from the private sector, while others may neglect the issue altogether. Table 4 provides an overview of the availability of contraceptive methods in clinics as described by providers.

**Table 4:** Quotes about availability of contraceptive methods

*“I only have two contraceptive methods here in the village: the microgynon, the femulin, and the condom”.* (P4, midwife) *“We have the microlut and the microgynon Pills, condoms, IUDs and Depo-Provera (hormonal injections)”.* (P7, midwife) *“Condoms have not been available for the past 3–4 years. Women ask for emergency pills, which are not always available, as many women use them if their condom accidently breaks down”.* (P11, midwife) *“The mini pills have not been available for a long period of time. Condoms have not been available since 2014”.* (P12, nurse) *“Of course contraceptive methods are not available here. Femulin (an oral contraceptive) has not been available here since August 2021…”* (P8, midwife) *“They used to provide us with vaginal suppositories in the past, and women used these suppositories with the condom to increase its effectiveness or to protect from pregnancy if the condom broke, but now they are not available”.* (P9, midwife)	

### Subtheme 5: suggestions for improvement

A main recommendation considered as crucial for enhancing FP services, as highlighted by the majority of providers, is to ensure uninterrupted availability of all FP methods. This should be coupled with making these services free for insured women and offered at minimal or no cost for non-insured women. Additionally, providers emphasized the importance of maintaining a consistent presence of healthcare professionals on a regular basis, particularly in urban clinics, as a crucial step toward improving FP services. Furthermore, providers suggested the need for updating competencies and providing ongoing training for healthcare professionals to enhance the quality of care in FP services.

*“Suggestions only provide us with resources and increase public awareness resources”.* (P6, physician)

*“The service must be available daily. If you want to increase the family planning service, you need to ensure that the service is available and that the doctor is present daily. Maybe this is difficult in the villages. but in the city, service should be available daily”. P8*.

*“We need to make all family planning methods available. This is the most important suggestion.”* (P11, midwife).

*“I hope that all contraceptives do not need insurance. Many people do not have insurance and are too poor to pay 1000 shekels to get insured. If all contraceptives are free, it will change many things, and demand for them will increase.”* (P13, physician).

Midwives suggested expanding their scope of practice by allowing them to perform IUD insertions. They argued that, unlike physicians who serve multiple clinics, midwives are consistently present on a daily basis in these clinics. Additionally, they recommended introducing and training on new methods such as implant, as there is a demand for this method among women. Furthermore, midwives working in clinics located in remote areas shared insights into the challenging and primitive process they currently employ to sterilize equipment, involving the use of vinegar, lemon, and carbonated soda. They suggested equipping clinics with basic equipment such as lights and sterilization machine.

*“There is no light. We need a better sterilization device.”* (P12, nurse).

*“During sterilization or drying, water drops on equipment, and it becomes rusty. So we use primitive methods to soak equipment like vinegar, lemon salt, and carbonated soda and remove the rust from it.”* (P12, nurse).

### Theme 3: structural barriers to FP services

As we dig more into the barriers to FP services, three sub-themes have emerged, as reported by healthcare providers.

### Subtheme 1: COVID-19’s negative effect on FP services

The COVID-19 lockdown had negative impact on FP services, as it resulted in the closure of all primary healthcare clinics, except for essential services like vaccination and treatment of sick patients/patients with chronic health conditions. Providers explained that FP services represent a cumulative achievement in terms of expertise and service provision. It took years to build trust within local communities, and women gradually began to access these services. Unfortunately, the closure of clinics due to the pandemic has disrupted this progress, essentially resetting these services to their starting point.

*“During the Lockdown, which lasted from March to August 2019, people became afraid to come to clinics. Family planning services almost did not exist during this period. We were only doing vaccination and giving medications to patients, and thus COVID-19 was a big barrier to family planning services.”* (P5, nurse).

### Subtheme 2: providing FP services with empty hands

#### Accessibility to FP methods

A major obstacle reported by providers is the inaccessibility of FP services for women due to several reasons. Firstly, these services are often offered on interrupted schedules, typically one to two days per week or month in clinics, rather than on a daily basis. This irregular availability does not always suit the schedules or health conditions of women seeking the services. Consequently, some women may opt for private providers or may even neglect the important matter of FP altogether.

*“We provide family planning services two days each week. So, when a woman comes for the service on Sunday, we ask her to come back on Monday. If she came on Tuesday, we asked her to come back on Wednesday.”* (P7, midwife).

Secondly, women, whether insured or uninsured, are required to pay for FP services. While these fees may seem small amounts to some, for many families experiencing financial difficulties, they can represent a significant financial burden. Furthermore, providers noted that women not only have to cover the cost of the contraceptive method they intend to use but also pays for each step they take to reach the service, including file opening, laboratory tests, ultrasounds, physician consultations, and the cost of the chosen FP method. According to providers’ reports, the cumulative expenses associated with each of these steps can nearly match the cost of obtaining the same service in private clinic.

*“One of the obstacles is that the service has become paid for, after being free. Now, even if the woman is insured, she has to pay 10 Shekels to open her file, she has to pay for the ultrasound; she has to pay to receive the contraception method.”* (P8, midwife).

Thirdly, the physician may not come on the assigned day of FP to the clinic. Physicians may be absent due to reasons such as being off work or attending educational or administrative meetings. Moreover, there is a shortage of female physicians, who are the preferred choice of women seeking FP services. Additionally, the existing female physicians may not always possess the skills to offer all the necessary FP services.

*“Because there is a shortage of female doctors, if the female doctor goes there, she is not trained to install the IUD, and women there prefer IUDs”* (P8, midwife).

*“The doctor is not always present. There are always insufficient doctors. The clinic would be frequently canceled. In some clinics, doctors come twice /month.”* (P9, midwife).

Fourth, many contraceptive methods are not available in clinics. Providers reported deficiencies and a lack of availability of several contraceptive methods in their clinics, such as condoms, mini-pills, IUDs, vaginal suppositories, emergency pills, and hormonal injections. (Table 4)

*“Women ask for emergency pills, which are not always available, as many women use them if their condom accidently breaks down.”* (P11, midwife).

*“Women ask for IUDs and they are shocked when they know that IUDs and pills are not available in our clinic.”* (P11, midwife).

*“The mini pills not been available for a long period of time. Condoms have not been available since 2014.”* (P12, nurse).

#### Structural and disposal deficiencies

Healthcare providers have highlighted many structural and infrastructure deficiencies within clinics, which hinder the provision of FP services. Many clinics struggle with issues such as inadequate space, insufficient privacy, absence of a sink within the examination room, the unavailability of portable lights for procedures like IUD insertion, a shortage of cleaning and sterilization facilities, and even a shortage of basic supplies like bed sheets. Several providers noted that the same room is often utilized for multiple purposes i.e. FP, antenatal care and pediatric clinics, further exacerbating these challenges. Table 5 provides examples of the Structural and disposal deficiencies in clinics as described by providers.

**Table 5:** Quotes about structural and disposal deficiencies

*“There is no place to wash my hands, so I rub my hands with alcohol, then I go to the delivery room to wash them.”* (P13, physician) *“I may use a phone light because we have no light in the clinic, no sink or toilet in the room.”* (P13, physician) *“In this clinic, the service of IUDs was stopped, because there is no bathroom, no place to wash the equipment. We only give tablets.”* (P8, midwife) *“There is no privacy. There is no private room for FP. Look at my room here. Pregnancy care, the pediatrician, and I in the same room; I have this room for family planning, pregnancy care, and to treat children at the same time.”* (P9, midwife) *“We are supposed to wear sterile gloves when installing an IUD. But there are no sterile gloves here. The doctor finds a way and uses certain mechanisms to install the IUD under sterile technique with someone helping her ”.* (P2, midwife)	

### Subtheme 3: Context, and cultural barriers

Despite the noticeable progress in women’s and families’ attitudes towards and acceptance of FP methods over the past two decades, certain cultural beliefs, misconceptions, and insufficient awareness among women continue to obstruct their access to FP services. For instance, providers have reported that some women still hold the belief that contraceptive methods can lead to health issues such as backaches, weight fluctuations (either thinness or weight gain), or even infertility.

*“We have misconceptions in this community about long-term infertility. People aren’t willing to believe that, for example, pills cannot cause infertility.”* (P6, physician).

*“Some women would complain that they became thin or fat or had backache due to the IUD. This is a common myth among women related to IUDs.”* (P11, midwife).

Moreover, some providers reported that the decision to use contraception is mainly the decision of the husband and sometimes the family and is mainly due to the financial hardship of the family, or if the woman is very sick.

*“Because she does not want to use hormonal methods, we offer her condoms, and the husband does not want to use condoms. We explain to her that condoms are safe a high success rate, and she would reply that her husband does not want to use them.”* (P5, nurse).

Providers reported that some women refrain from using contraceptives for various reasons. These include the desire to have more children in the belief that it strengthens their marriage, the hope of having a boy if they already have girls, and concerns that contraception use may conflict with their Islamic beliefs.

*“I need to have more children so that my husband will not get married again. He will not be able to pay for his second marriage, and he will continue to financially support us.”* (P13, physician).

## Discussion

This study sheds light on key factors influencing the provision of FP services and provides insight into potential quality improvement interventions based on the perceptions of service providers. The study highlights the variation in knowledge, training, and experience of FP service providers. Although there are MoH guidelines on types and contraindications of different contraceptive methods, the service provision varies widely and depends largely on the personnel working at each clinic.

Physicians and midwives indicated that FP was included in their pre-service curricula. Some midwives and physicians reported that their FP pre-service preparation lacked adequate clinical training. Local bachelor midwifery curricula, which are university-based, include FP education and clinical training. However, universities face challenges in securing clinical placement for midwifery students, as all midwifery programs prefer clinical training for FP in UNRWA clinics where midwives effectively lead FP services. This challenge may result in some midwives graduating with inadequate clinical training in FP as noted by few midwives in our study. Despite receiving education at universities, midwives are not efficiently utilized to provide a wide range of sexual and reproductive health services particularly FP due to absence of law to protect their practice as noted by some midwives. On the contrary, some FP clinics were staffed with nurses who are not sufficiently trained to provide FP services.

In this study, midwives seemed to be more efficient and confident about their responsibilities, despite their role is primarily restricted to counseling and assisting physicians in the delivery of FP methods. Unlike some countries in the region like Sudan, Tunisia, Morocco and Oman where midwives act as primary providers for sexual reproductive health services, midwife’s role in Palestine is limited to pregnancy, labour and delivery [10]. Moreover, during the pandemic, midwives were deployed away from maternal health services and primary healthcare centers were mainly loaded with COVID 19 activities [20]. On the other hand, physicians and nurses reported learning by observing their colleagues or through personal practice, which is not always based on solid science.

Further, inadequate updated in-service competency-based training contributes to misconceptions and gaps in evidence-based practice, as evidenced by observations from certain interviews.

False information transferred to beneficiaries by their service providers is often difficult to change over time and can prevent them from using modern contraceptive methods. Misconception about hormonal contraceptives like fear of infertility is a well-recognized barrier to their use [21–23]. For example, a few providers demonstrated bias against hormonal methods, while others reported that they did not try to encourage women to switch to modern contraception if they were already using traditional methods like abstinence during fertility days. This situation is further complicated by the negative influence of husbands on women’s decisions to use contraception, as reported by some providers. Therefore, it is necessary that service providers receive comprehensive training to deliver proper counseling and education. This includes information on the effectiveness and failure rates of both modern and traditional contraceptive methods, particularly addressing issues such as advancing age or irregular menstrual periods. Moreover, counseling should go beyond individuals to include the social network of women, mainly the husbands. Research indicates that involving men in FP counseling can significantly increase the use of contraceptive methods [24–27]. In-service competency-based training is an effective means for enhancing the capacity of service providers. This occurs through onsite learning coupled with hands-on practice, enabling the development of skills and correction of myths and misconceptions [28–30]. The on-site training does not mandate the release of service providers during their working hours, thus avoiding disruptions to service delivery [28–30].

Accessing health facilities and services has always been challenging for Palestinians residing in the West Bank, primarily due fragmented areas separated by road obstacles, closures, checkpoints and walls resulting from occupation [31]. After the ongoing war on Gaza, which began in October 2023, hostilities and aggressions have markedly escalated in residential areas of the West Bank. Movement restrictions persist among various cities, posing significant challenge and becoming nightmare for Palestinians. According to the latest report from UNFPA, 73,000 pregnant women in the West Bank face challenges in accessing healthcare and delivery facilities [32]. Likewise, women face challenges in accessing FP services, which are distributed across cities, rural areas and camps throughout the West Bank.

Unequal access to and varying quality of FP services contribute to inequities and variations between regions. A commonly cited barrier to providing modern contraceptive methods, mainly LARCMs, is the scarcity of training opportunities [33, 34]. The lack of systematic and regular in-service training is a potential explanation for why the relocation or turnover of service providers could lead to interruptions in the continuity and quality of services. For instance, a midwife reported a decline in the use of IUD use following the retirement of a physician at their clinic, who was not replaced by adequately trained personnel.

A prominent obstacle to FP services reported by the providers, is the interrupted schedule and limited availability of physicians. This, coupled with narrow scope of midwives’ roles, hinders women’s access to FP services at convenient times. Indeed, some midwives expressed disappointment in this study due to the limitations in their scope of practice, despite being the only trained providers available at the clinic on a daily basis. It is noteworthy that the provision of FP services is a core competency for midwifery practice according to international midwifery curriculums standards [35]. However, midwives face many challenges that hinder their capacities to deliver high-quality FP services. These obstacles include inadequate knowledge/training, misinformation and myths, insufficient regulations, lack of resources, inadequate professional support and poor working environment and salaries [22, 36–39]. Expansion of midwives’ roles beyond the traditional provision of services has been recommended to improve access to FP services [36]. This role is reinforced by the fact that midwives usually spend the longest time with women and hence can discuss the needs and requests of women in a culturally sensitive manner [40]. The expanded role of trained midwives in the provision of postnatal LARC, like implants is usually positively received by women, midwives and their colleagues [41, 42]. On the other hand, some midwives raise concerns about expanding the scope of their practice due to insufficient training and burden of work overload [43]. Expanding the role of midwives’ role in FP services beyond counseling is a wise step, considering the shortage and turnover of Physicians. However, such expansion requires the development of an advocacy plan. This plan should include mobilizing political support, formulating and amending policies, raising public awareness, and implementing effective capacity building and supervision measures.

Most service providers reported that supervision primarily focused on administrative tasks rather than providing technical guidance. While work protocols exist in the form of announcements or information disseminated during monthly administrative meetings, there is a major gap in the provision of technical supervision. This directly affects the quality of healthcare. Supportive supervision of service providers is as important as training, as it aids in continuous professional development. It bridges the gap between knowledge and practice, enhances skills and confidence, and improves the performance of providers [44–46]. This should be conducted in a professional and respectful way to ensure that service providers feel comfortable reporting problems and addressing needs for improvement. This approach fosters a positive work environment and enhances outcomes for the benefit of beneficiaries [47].

In addition to the shortage of skilled human resources, context-specific barriers such as physical infrastructural deficiencies significantly hinder the fulfillment of FP demand. These include factors such as the accessibility and amenities of a health facility, as well as the availability of FP commodities [23, 45, 48–51]. Our study has identified similar challenges, including but not limited to inadequate space, inadequate privacy, shortage of sterilization tools, limited options of contraceptive methods, and recurrent and sometimes prolonged, stock-outs of different contraceptive commodities.

The forecasting and procurement of FP supplies follow similar procedures as for essential medicines at the MoH. One of the challenges is the lack of utilization of demographic data in forecasting. Quantifying FP commodities relies partly on estimated needs gathered from points of service delivery, but mainly on consumption data, which is not always accurate. The distribution of these commodities is mainly influenced by the consumption patterns, which plausibly explains why some clinics experience more challenges by stock-outs and sometimes overstocks. There is no universally accepted gold-standard forecasting method, highlighting the need for a standardized approach that effectively fulfills various aspects such as market size estimation and demand [52]. An additional challenge arises from the procurement process, which is completed through a bidding system. Sometimes, commodity suppliers may fail to submit a proposal or adhere to shipment deadlines, resulting in delays in distributing these supplies to health facilities.

## Conclusion

In conclusion, access to and quality of FP services in Palestine are faced with a complex set of inadequate facilities. It is crucial to meticulously address both community-related and health system factors to enhance the fulfillment of FP needs and reduce unintended and closely spaced pregnancies. A collaboration with higher education institutions should enhance the complexity of providing culturally sensitive and appropriate care, tailored to women’s individual needs. Implementing regular, competency-based, in-service training for midwives, physicians and nurses will enhance the capacities of healthcare providers to provide high-quality comprehensive FP services. Policymakers should invest in the development of necessary laws and regulations regarding FP services, promoting a comprehensive and holistic approach to such services. This includes formulating supportive policies for midwifery practice, capacity building of human resources and maintaining security of FP commodities.

### Strengths and limitations

The study’s limitations are firstly related to those interviews were restricted to governmental providers as our focus was on gaining a comprehensive understanding of the barriers and opportunities related to FP services within the MoH context. It is worth noting that FP services provided by other sectors may encounter different barriers, which is beyond the scope of this study. Secondly, interviews were conducted at limited number of clinics. However, researcher ensured to select clinics with high volume of FP service provision. These clinics were located in diverse areas across the West Bank, including cities, rural areas and refugee camps, spanning various governorates in the north, middle and south. This selection aimed to include a diverse beneficiary population. Thirdly, despite the small sample size, the findings are relevant to other MoH clinics facing similar constrains. Furthermore, it is important to note that the researchers did not intend for generalization; rather, the aim was to thoroughly examine and understand barriers and existing opportunities for enhancing FP services at the public sector.

### Electronic supplementary material

Below is the link to the electronic supplementary material.

Supplementary Material 1

Supplementary Material 2

Supplementary Material 3

## Data Availability

Data is provided within the manuscript or suplementary information files.

## Abbreviations

FP: Family Planning
oPt: Occupied Palestinian territory
MoH: Ministry of Health
SDGs: Sustainable Development Goals
LARCMs: Long-lasting Reversable Contraceptive Methods
SRHR: Sexual Reproductive Health and Rights
SRH: Sexual Reproductive Health
UNRWA: United Nations Relief and Works Agency
PHC: Primary Health Care
UNFPA: United Nation Population Fund
