# Supplementary material for: Perceived barriers and opportunities of providing quality family planning services among Palestinian midwives, physicians and nurses in the West Bank: a qualitative study
Source: BMC Health Serv Res. 2024 Jul 9;24:786. doi: 10.1186/s12913-024-11216-4 (PMC11234620; doi:10.1186/s12913-024-11216-4)
Supplement: Supplementary file 1 — Supplementary Material 1 [file 12913_2024_11216_MOESM1_ESM.pdf]

Additional file 1

Interview guide (English)

Please obtain the following information from each participant:

Background information of each participant.

1. Personal: age, sex.
2. Governorate, name of clinic (PHC), position.
3. Professional background: Education (midwifery, medicine, nursing), Type of education (bachelor/Masters, specialized..?), year of graduation.
4. Experience: years of experience. Since when been working in this PHC?

Education/training/practices

1. Type of education/training did you receive on FP as part of your formal education at university?
2. Training on FP received after being employed regarding FP services? (Probe to get further insight).
3. Do you feel competent in counseling women on contraception advice?
4. What FP methods you are competent to offer for women?
5. What FP methods you are not trained enough to offer for women?
6. What FP methods you need further training on to feel confident to practice?
7. Are you allowed to provide FP services on your own?
8. Are you covered by your employer/MoH in case of complications?
9. What are the major obstacles/barriers you face during delivering services to your clients in this center?
10. What equipment you require for FP services at your clinic and are deficient or not available?
11. What FP methods are available at your clinic? Are those always available or there are periods of stock-out? What methods you do not get regularly? Or usually unavailable?
12. Do you often face problems with availability of FP methods/equipment? How do you manage when you have deficiencies? What would you tell women who come to the PHC requesting a FP method that is not available?
13. Do you often encounter overstocking of any of the family planning methods? If yes, what do you think might be the cause?
14. When do you usually talk to women about FP options (postnatal visit, preconception, at well-baby clinic/vaccine)? What challenges do you face then (time, space)?
15. How necessary are FP services to women attending your clinic?
16. What is the biggest challenge that you face in FP services?
17. How you evaluate FP services in your clinic?

Facilitators

1. Do you have clinical specific guidelines and protocols that guide you into FP services? When these protocols/guidelines were issued? (Year). Were you oriented/trained on these guidelines? (Formal/on-job training/informal).
2. Did you receive a hands-on training in IUD or implant insertion? At clinic or workshop?
3. What kind of monitoring/supervision/support do you receive at your clinic regarding FP services? If any, does this helps you to provide quality FP services?

Suggested solutions/strategies

1. Suggestions/solutions to strengthen, enhance, and upscale FP services at your clinic? (Probe around guidelines, training, expanding scope of midwives)
2. Aspects need improvements/changes?
