# Supplementary material for: Perceived barriers and opportunities of providing quality family planning services among Palestinian midwives, physicians and nurses in the West Bank: a qualitative study
Source: BMC Health Serv Res. 2024 Jul 9;24:786. doi: 10.1186/s12913-024-11216-4 (PMC11234620; doi:10.1186/s12913-024-11216-4)
Supplement: Supplementary file 2 — Supplementary Material 2 [file 12913_2024_11216_MOESM2_ESM.docx]

Appendix 1: Example of data extract containing units of data and line-by-line coding

| Data extract units of data | Early descriptive codes/line-by-line  coding |
| --- | --- |
| Participant #9  Well, regarding the challenges from the Ministry of Health, they are the same challenges in all clinics. There's no privacy, no separate room for family planning (FP). In most cases, only a very few clinics have them. For instance, look at my room here – I and the antenatal care are in the same room. I and the pediatrician are in the same room. There's no privacy, no dedicated FP room. Unfortunately, the ministry doesn't establish clinics; they take existing buildings from the local community, and not all of them are properly equipped with separate room for FP, separate room for antenatal care, separate room for children – all in the same room at the same time.  Are there any other challenges?  Well, sometimes when I want to work with her (a woman), I have to ask the doctor to please wait outside until I'm done with the woman.  What other challenges do you face?  This is the most important thing, what do you want to know more?  Generally speaking, the challenges you mentioned about providing the service are related to the doctor.  Yes, the doctor isn't always available. I mean, there's a constant shortage of doctors. We're always short on doctors. Sometimes, they send you a doctor once a month or twice a month, for example. Our clinic is supposed to have a weekly schedule, but a doctor rarely comes 4 times a month." | no privacy  no separate room for FP (space)  only a very few clinics have separate rooms (space)  FP and antenatal in the same room (space)  FP and child clinic in same room (space)  no privacy  no special room for FP services (space)  No new buildings (space)  Clinics in existing building (space)  Local communities offer building for clinics  Rooms not properly equipped/separated  All services occur in one room (space)  Cannot work with women in privacy  Reorganizing staff might enable privacy to conduct FP  Doctors not always available  Shortage of doctors  Doctors do not attend clinic per schedule  Doctor come 1-2 times/month |
