# Supplementary material for: Perceived barriers and opportunities of providing quality family planning services among Palestinian midwives, physicians and nurses in the West Bank: a qualitative study
Source: BMC Health Serv Res. 2024 Jul 9;24:786. doi: 10.1186/s12913-024-11216-4 (PMC11234620; doi:10.1186/s12913-024-11216-4)
Supplement: Supplementary file 3 — Supplementary Material 3 [file 12913_2024_11216_MOESM3_ESM.docx]

Appendix 2: Example of development from colliding codes to sub-themes

| Codes and collated data | Initial sub-themes |
| --- | --- |
| Privacy  Space  Not enough room for various services  Local communities offer building for clinics  Lack of equipment  Barriers related to healthcare providers:   - Shortage of Physicians - Irregular and inadequate physicians’ attendance to clinic | Structural barriers to FP services  Barriers related to availability of equipment  Barriers related to health care providers |
